# Tangible nanocomposites with diverse properties for heart valve application

**DOI:** 10.1088/1468-6996/16/3/033504

**Published:** 2015-05-20

**Authors:** Muthu Vignesh Vellayappan, Arunpandian Balaji, Aruna Priyadarshini Subramanian, Agnes Aruna John, Saravana Kumar Jaganathan, Selvakumar Murugesan, Hemanth Mohandas, Eko Supriyanto, Mustafa Yusof

**Affiliations:** 1 IJN-UTM Cardiovascular Engineering Centre, Faculty of Biosciences and Medical Engineering, Universiti Teknologi Malaysia, Johor Bahru 81310, Malaysia; 2 Rubber Technology Center, Indian Institute of Technology, Kharagpur, 721302, India; 3 Department of Biomedical Engineering, University of Texas Arlington, Texas, TX 76019, USA

**Keywords:** nanocomposites, heart-valve, hemocompatiblity, anti-calcification, mechanical-strength

## Abstract

Cardiovascular disease claims millions of lives every year throughout the world. Biomaterials are used widely for the treatment of this fatal disease. With the advent of nanotechnology, the use of nanocomposites has become almost inevitable in the field of biomaterials. The versatile properties of nanocomposites, such as improved durability and biocompatibility, make them an ideal choice for various biomedical applications. Among the various nanocomposites, polyhedral oligomeric silsesquioxane-poly(carbonate-urea)urethane, bacterial cellulose with polyvinyl alcohol, carbon nanotubes, graphene oxide and nano-hydroxyapatite nanocomposites have gained popularity as putative choices for biomaterials in cardiovascular applications owing to their superior properties. In this review, various studies performed utilizing these nanocomposites for improving the mechanical strength, anti-calcification potential and hemocompatibility of heart valves are reviewed and summarized. The primary motive of this work is to shed light on the emerging nanocomposites for heart valve applications. Furthermore, we aim to promote the prospects of these nanocomposites in the campaign against cardiovascular diseases.

## 1. Introduction

Cardiovascular disease is a fatal disease and the health data obtained from more than 190 countries show that heart disease is the number one global cause of death, with 17.3 million deaths every year according to the latest 2015 health report. This number is predicted to increase to more than 23.6 million by the year 2030 [1]. As per the Euro Heart Survey, aortic valve disease constitutes 44.3% of all cardiac valve failures [2]. This makes the aortic valve replacement (AVR) operation the second most common cardiac operation after coronary artery bypass grafting [3]. Even though there have been various advancements in AVR in terms of valve design, surgical techniques and concomitant medication, the available prostheses are still not able to meet essential characteristics such as durability in the case of bioprosthetic heart valves (BHVs) and thrombogenicity for mechanical heart valves (MHVs).

**Figure 1.**
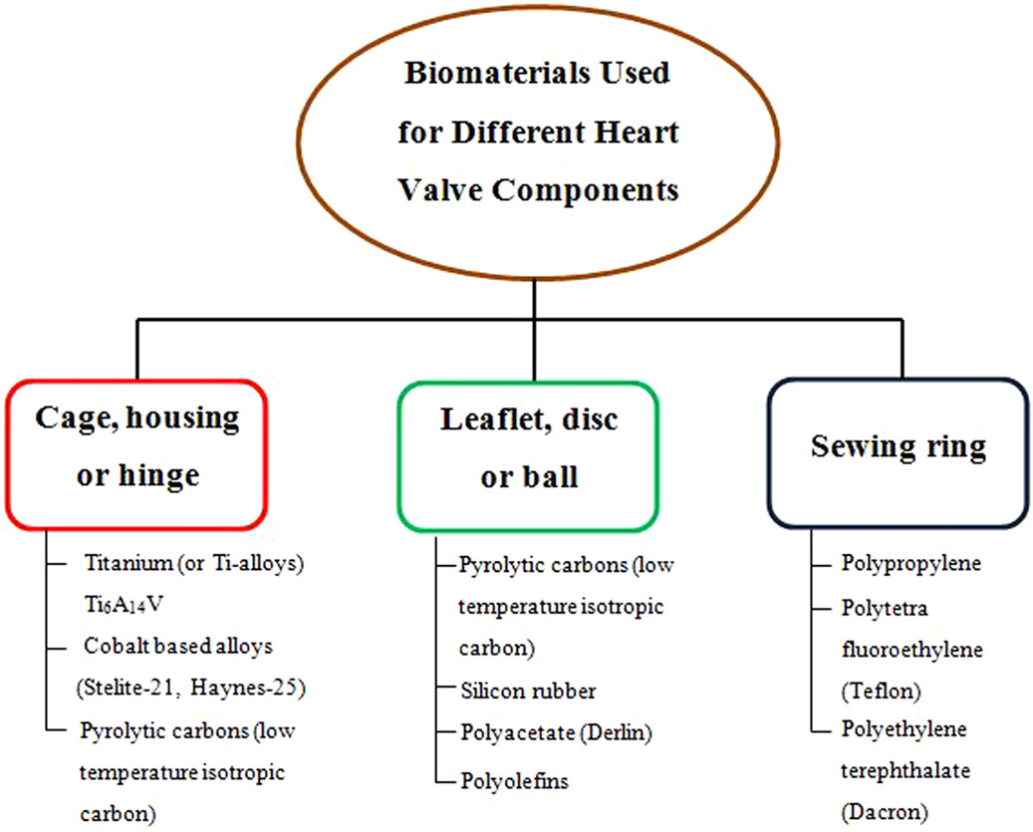
Biomaterials used for different heart valve components.

The development of cardiovascular heart valves demands an in depth knowledge and understanding of the mechanical and surface properties of the materials used for heart valve design. Thus, the development of a substitute heart valve mimicking the performance of the natural heart valves exactly remains as a herculean challenge for biomedical engineers to decipher. The ideal artificial heart valve must have certain clinical and engineering features to perform efficiently. A viable biomaterial which is completely functional and blood compatible is the most vital factor to be considered when designing and developing prosthetic heart valves. There is a set of characteristics that have to be contemplated for selecting a suitable prosthetic heart valve material. For example, the surface of the heart valve material should cause minimal or no damage to the circulating blood cells and to the surrounding endothelial tissue of the cardiovascular structure. The biomaterial utilized must exhibit excellent resistance to mechanical and structural wear. The possibility of platelet and thrombus deposition on the biomaterials should be small. The material used should be non-degradable in the physiological environment and must neither absorb blood constituents nor release foreign particles into the blood stream. The heart valve biomaterial should also be non-hemolytic, non-thrombogenic, non-infectious, non-immunogenic, non-inflammatory and non-calcifying. The two commonly used types of heart valves are MHVs and BHVs. Currently, few bioengineering materials are suitable for MHVs and these have limited applications. Figure 1 depicts the various materials used for the components of MHVs [4].

Synthetic leaflet heart valves (SLHV), including polyurethane (PU) valves, have been studied to develop a best alternative to currently available cardiac valve prostheses. This has be achieved by combining the more durable MHVs and improved hemodynamic BHVs to overcome their limitations. Desirable physical properties, affordable manufacturing and the same flow dynamics as human heart valves made PU valves a plausible substitute for mechanical and tissue prostheses. Conversely, they were not used as heart valve replacements as they suffered premature failure because of their sub-optimal design and the lack of a durable polymer [5]. A few synthetic materials, such as silicon rubber, polyolefin, polytetrafluoroethylene (PTFE), poly(styrene-block-isobutylene-block-styrene), ethylene propylene diene monomer (M-class) rubber, polyvinyl alcohol (PVA) cryogel and segmented PU elastomers have been tested as heart valve leaflets [6–10]. However, these materials did not have sufficient physical strength and the long-term biostability required for heart valve prostheses. Thus, an unmet demand exists for the development of new materials with enhanced properties for heart valve development. Recent trends and advancements in synthesis methods and structural modification, along with the emergence of novel technologies such as nanotechnology, have paved the way for the development of superior materials called nanocomposites which can be explored for heart valve applications.

Nanocomposites are materials which are produced by the mixture of components at a nanometer scale. They are composed of a minimum of two constituent materials which form the matrix or host plus a bolstering component called a nanofiller or guest [11]. It is a well known fact that the characteristics of a material change significantly when the size is substantially smaller, in the range of 1–100 nm. As they are quantum scale in size, they can serve as a connecting bridge between molecules in the polymer. This makes it possible for nanocomposites to display a different range of properties to regular microcomposites.

Nanocomposite materials may be either organic, inorganic or hybrid in composition [12]. Different examples of nanocomposites which are naturally available are bone, abalone shells and teeth. Inspired by the excellent properties of natural nanocomposites, different studies have been performed for producing synthetic nanocomposites. The synthetic nanocomposites were expected to have the same outstanding properties as natural nanocomposites [13, 14]. The technique which is used to create the nanocomposites is the determining factor for the performance of the synthetic nanocomposite material. For instance, different factors such as production technique, the nature of the nanofiller, the process used for nano-reinforcement and the type of reactions involved between the polymeric and strengthening constituents regulate the overall performance of the nanocomposite material [11]. There are different types of synthetic nanocomposites and they can be segregated into four categories which are clay-, carbon-, metal- and glass-reinforced [15]. Silicon and metal oxides (e.g. ZnO and TiO_2_) are used frequently as nanofillers due to their superior mechanical properties [16]. As the physical, chemical and mechanical properties of nanomaterials are far better in comparison to conventional biomaterials, they are putative choice for cardiovascular applications.

Even though there are various problems associated with heart valves, the leading problem is heart valve failure due to mechanical stress, calcification of the heart valve and blood compatibility issues [17]. Despite the fact that MHVs and BHVs have been used for more than fifty years, no appreciable clinical outcome has yet been attained. The National Institutes of Health (NIH) reported that the ten year mortality rate for valve replacements still varies from 30–55%, indicating the severity of the heart valve failure problem and emphasizing the need for further improvements in valve substitutes [18]. Thus, in this review the three vital issues mentioned above for conventional heart valves are analyzed thoroughly and the vital role nanocomposites can play in tackling these issues is summarized.

## 2. Potential nanocomposites for cardiovascular heart valve applications

Finding materials suitable for soft tissue replacement is a significant aspect for heart valve design and fabrication. There is a demand for a material that not only exhibits similar mechanical properties as the heart valve it is replacing, but also demonstrates improved life span, biocompatibility, and low thrombogenicity and degree of calcification. In a recently published work, the biomechanical properties of native and tissue engineered heart valve constructs were compared [19]. It was shown that the uniaxial tensile mechanical properties, the Young's modulus, ultimate tensile strength and strain at the ultimate tensile stress of human native heart valves, in the circumferential direction are 15 MPa, 2.6 MPa and 22 MPa, respectively [20]. Similarly, the same three mechanical properties of the native heart valve in the radial direction were found to be 2 MPa, 0.4 MPa and 30 MPa, respectively [20]. These values indicate that the modulus of elasticity and the ultimate tensile stress of heart valve leaflets are greater in the circumferential direction compared to the radial direction. On the other hand, a prominent difference was observed between human heart valves and animal heart valves such as porcine heart valves. The Young’s modulus, ultimate tensile strength and strain at maximum tensile stress of a porcine heart valve were found to be 7.78 MPa, 16.8 MPa and 10.80 MPa in the circumferential direction and 1.28 MPa, 11.60 MPa and 7.50 MPa in the radial direction, respectively [21]. This shows why animal heart valves are much weaker in comparison to human native heart valves and dictates that xenograft valve transplants in humans lack long-term durability. Hence, the use of nanocomposites holds great potential for circumventing this mechanical property bottleneck, since the mechanical property values of the nanocomposites are in the same range or have greater mechanical strength values compared to the native heart valve. Apart from the improved mechanical properties, the improved hemocompatibility and anti-calcification of nanocomposites allow us to envisage their application in cardiovascular heart valves. There are numerous nanocomposites which have emerged recently. Polyhedral oligomeric silsesquioxane-poly(carbonate-urea)urethane (POSS–PCU), bacterial cellulose (BC) with PVA, carbon nanotubes (CNTs), graphene oxide (GO) and nano-hydroxyapatite (nHA) nanocomposites have gained popularity as putative choices for biomaterials in cardiovascular applications owing to their superior properties. A succinct insight on these nanocomposites is given in the following before we discuss their putative role in heart valve applications.

**Figure 2.**
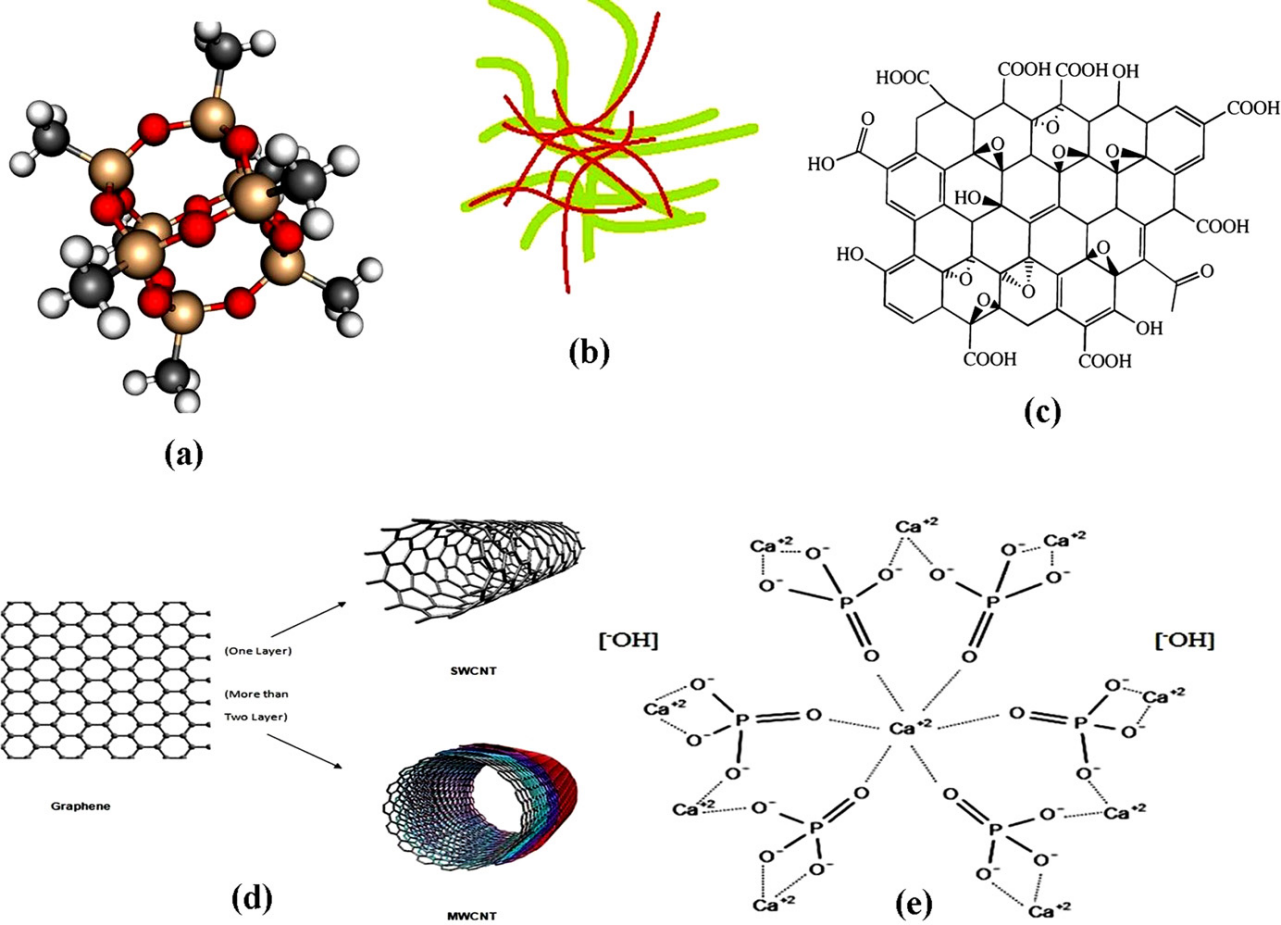
Structures of fillers in nanocomposites for heart valve applications.

POSS nanoparticles added to PCU, resulting in POSS–PCU, has been developed for various surgical implant applications. The chemical structure of the silsesquioxane family is defined as R_*n*_Si_*n*_O_1.5*n*_. Figure 2(a) shows the three-dimensional structure of POSS which consists of an inner inorganic framework of silicon (red) and oxygen (yellow) atoms, externally covered by atoms of organic groups (grey) and hydrogen atoms (white). This forms the cage structure of the POSS. The inclusion of unique nanoscale POSS moieties in PCU functions as a cross-linking agent and tailors the physicochemical properties of this nanocomposite polymer in comparison to conventional constituents [22]. POSS–PCU was utilized for many vital biological applications such as the world's first artificial trachea, lacrimal duct conduits and lower limb bypass grafts, all of which are about to enter clinical trials [23–25]. Thus, the application of the POSS–PCU nanocomposite can be further extended for heart valve development.

BC is a viable, sustainable and biodegradable nanofibrous material. The diameter of BC fibers ranges from 40–70 nm and these fibers demonstrate various unique properties such as high purity, a better degree of polymerization and excellent crystallinity, thereby giving them a high strength and modulus [26–28]. The smaller fiber diameter of BC confers a higher surface area and its porous structures facilitate the retention of a large amount of water. BC also has good thermal stability, outstanding environmental biodegradability and exceptional biocompatibility [26–28]. Owing to its excellent properties, BC is used in a wide range of applications such as artificial bones, skin, blood vessels, etc [29, 30]. PVA is a hydrophilic biocompatible polymer. It has the desired characteristics for biomedical applications. PVA can be converted into a solid hydrogel with better mechanical properties by physical crosslinking, using freeze–thaw cycles. Thus, PVA combined with BC results in biocompatible nanocomposites with synergetic properties. Figure 2(b) shows the PVA–BC structure where the green strains are BC and the brown strains represent the hydrophilic PVA strains which are combined to form a PVA–BC nanocomposite. This PVA–BC has a wide spectrum of mechanical properties and it can be tailored to possess mechanical properties almost identical to those of cardiovascular tissues such as heart valve leaflets. Hence, we can exploit the superior properties of PVA–BC for cardiovascular heart valve applications.

Graphene is a one atom layer of carbon and has received enormous attention for its novel properties and plausible applications in the field of materials science since it was first reported in 2004 [31]. Graphene-based materials can be synthesized in different forms such as GO, reduced GO and exfoliated graphite, which guarantees its scalable production and supply for a variety of applications. GO is an oxidized graphene derivative which can be used as a substitute or precursor for graphene materials owing to its high dispersibility and processability in aqueous environments [32, 33]. It is produced from mineral graphite flakes via a thermal oxidation method. Figure 2(c) shows the structure of GO. GO has various functional groups such as epoxy, hydroxyl and carboxyl groups, which make it strongly hydrophilic. This makes it a good candidate for applications in biomolecules, drugs and inorganic nanoparticles [34–37]. Moreover, graphene-based materials confer immense application prospects in biomedicine. Hence, GO is a putative candidate for the development of prosthetic heart valves.

A CNT can be considered as a seamless hollow tube consisting of a graphite sheet where, depending on the number of graphite sheet layers, the CNTs can be classified into single-walled CNTs (SWCNTs) and multi-walled CNTs (MWCNTs) [31, 38]. A SWCNT is a single molecular nanomaterial, composed of only one layer, a rolled single sheet of graphite (graphene) forming a molecular cylinder. Its diameter and length vary in the ranges of 0.75–3 nm and l–50 nm, respectively. A MWCNT consists of more than two layers of curly graphite sheet, with diameters of 2–30 nm and some even more than l00 nm. The distance between each layer is approximately 0.42 nm. Figure 2(d) shows CNTs. CNTs are widely used in various biomedical applications such as biosensors and diagnostic agents to visualize cancer cells etc, due to their biocompatible properties [39, 40]. Therefore, CNTs are also a potential candidate for the development of cardiovascular heart valves.

nHA is one of the potential ceramic nanofillers with a multitude of applications. It has a chemical structure of Ca_10_(PO_4_)_6_(OH)_2_ and is referred to as a calcium phosphate tribasic phase [41]. It is shown in figure 2(e). Recently, nHA has gained momentum as an nHA-based filler reinforcement for polymer matrices in biomedical applications such as tissue engineering and bone implants. HA reinforced polymer nanocomposites confer excellent biocompatibility in combination with enhanced mechanical properties such as improved tensile and impact strength. It was found that the inclusion of about 10% nHA by weight increases the stiffness and strength of the polymeric matrix [42].

In the following sections, three vital issues—namely mechanical strength, anti-calcification potential and hemocompatibility—will be addressed, citing the improvements facilitated by using nanocomposites.

## 3. Nanocomposites for improving the mechanical strength of prosthetic heart valves

From the perspective of structural and mechanical properties, it is expected that when the heart valve is made up of a fiber reinforced composite material which mimics the natural heart valve structure and properties, it will minimize perforations, leaflet stresses and tears. Commonly used materials, such as poly(tetrafluoroethylene), polyvinyl chloride, segmented poly(urethane), silicon rubber and poly(ether urethane urea), have drawbacks such as fatigue, short life-span and durability [43]. The incorporation of novel nanocomposite materials in heart valves to overcome these drawbacks is discussed in the following.

Wan *et al* ascertained that BC fibers are very strong and can be used for the development of cardiovascular heart valves [44]. In their work, a nanocomposite was synthesized by combining BC networks and calcium-deficient HA powders and the nanocomposite produced was then characterized. The nHA nanoparticles were prepared via a wet chemical precipitation method, commencing from aqueous solutions of calcium nitrate and di-ammonium phosphate salts. Energy-dispersive spectroscopy showed that the prepared HA was calcium-deficient HA. BC–HA nanocomposites were then prepared through the addition of carboxymethylcellulose (CMC) to the bacteria culture medium. nHA nanoparticles were then added and remained suspended in the culture medium during the formation of cellulose nanofibrils. The maximum gel thickness was observed after 21 days of bacteria cultivation. X-ray diffraction (XRD) results showed the variation in crystallinity among the materials involved in the formation of the nanocomposites. The inorganic and organic bonds between the HA and BC were ascertained using attenuated total reflectance Fourier transform infrared spectroscopy. Scanning electron microscopy (SEM) and atomic force microscopy (AFM) analyses confirmed the formation of networks and fibers with minimum diameters corresponding to BC synthesized in the presence of CMC. Qualitative analysis was implemented to study the orientation distributions and Feret diameters for networks of BC and BC–CMC. Thermogravimetric analysis showed that the quantity of the mineral phase was 23.7% of the total weight of the nanocomposite. These results indicate that there would be formation of new networks and fibers with a smaller diameter resulting in a good increase in the mechanical strength of the resultant nanocomposite. Thus, this nanocomposite with properties mimicking cardiovascular tissues can be considered as a potential candidate for synthetic replacements of cardiovascular tissues such as heart valves.

Likewise, in a work by Millon and Wan, the effect of the inclusion of BC in PVA and the mechanical properties of the resulting nanocomposite was studied [45]. Surprisingly, the resultant nanocomposite was found to possess excellent mechanical properties, similar to those of cardiovascular tissues such as heart valve leaflets. The stress–strain characteristics of porcine aorta were matched by at least one type of PVA–BC nanocomposite in either the circumferential or axial tissue directions. A PVA–BC nanocomposite with same mechanical characteristic as natural heart valve tissue was developed in this study. The relaxation characteristics of samples were also studied. The result shows that PVA–BC relaxes at a faster rate and has a lower residual stress compared to the natural tissues it replaces. Hence, the PVA–BC composite can be promoted as a promising material for cardiovascular heart valve applications.

Alongside the above studies, Kidane *et al* investigated the mechanical properties of POSS–PCU, such as tensile strength, tear resistance and hardness and then compared them with normal PCU [46]. In addition to mechanical properties, surface properties such as contact angles and platelet adhesion resistance were assessed. It was found that POSS–PCU (hardness 84.0 ± 0.8 Shore A) exhibited considerably greater tensile strength (54 ± 3 and 56 ± 4 N mm^−2^ at 25 °C and 37 °C, respectively) in comparison to normal PCU (34 ± 2 and 29 ± 3 N mm^−2^). Elasticity properties such as tensile strength, elongation at break, and Young’s modulus were found to be higher in the case of POSS–PCU than the control PCU at 25 °C and 37 °C. There was no significant difference observed in the tear strength between POSS–PCU and the control PCU at 25 °C; however, when the temperature was increased to 37 °C POSS–PCU displayed superior tear strength to PCU at a thickness of 200 *μ*m (63.0 ± 1.5 N mm^−1^).

Xu *et al* demonstrated that the strength and hardness of PCU increased with the inclusion of POSS particles into the polymer matrix [47]. The improved tensile strength and hardness of POSS–PCU was believed to be due to the inclusion of nanoscale POSS particles in the polymer matrix. The special property of the POSS nanocomposites is that they are held together by a strong intermolecular force between the nanocomposite molecules and neighboring molecules. In addition, a strong framework with shorter bond lengths exists between the nanocomposites, providing them with additional resistance to degradation. A POSS nanofiller will improve the material’s transition temperature (*T*_g_) at greater concentrations by reducing the distance between nanofillers. As a result of this, the molecular or segment rotation with respect to the reference polymer will be decreased thereby making the polymer more robust. The POSS-incorporated polymer’s mechanical properties were studied at a higher temperature and it was found that this mechanical property was retained. Hence, POSS–PCU may be considered as a putative choice for heart valve applications.

In addition to the studies on POSS–PCU discussed above, Ghanbari *et al* showed that the glass transition temperature (*T*_g_) was found to be almost same for both PU and POSS–PCU [48]. This is due to the fact that only a very low percentage of POSS was added to the POSS–PCU. The percentage of POSS addition was only 2%. Similarly, the stress–strain behaviors of both the POSS–PCU and PU were also nearly the same but the maximum tensile strength and elongation at the break were found to be smaller in PU than in POSS–PCU. Thus, this property of POSS can be exploited to enhance the long term durability of heart valves developed from POSS–PCU. Likewise, Petrovic *et al* demonstrated that the mechanical properties, such as maximum tensile strength and elongation at the break, were improved in the case of PU modified with POSS compared to the control PU [49].

CNTs have been characterized as having outstanding mechanical properties. Both SWCNTs and MWCNTs have a large Young’s modulus (1 TPa) because of their flexible hexagonal network of carbon atoms [50, 51]. Thus, CNTs have been added as bolstering agents in natural and synthetic polymer matrices. It was observed that in these nanocomposites, CNTs play an important role in enhancing the mechanical and structural properties of polymer composites. Poly (propylene fumarate) (PPF), which is a biodegradable polyester, is a good polymeric biomaterial used for tissue regeneration applications. It was found that the inclusion of SWCNTs into the PPF polymer improves the mechanical properties of PPF. The presence of very low concentrations of SWCNTs (less than 0.5 wt%) in the PPF polymer matrix significantly improves (up to two- to three-fold) the compressive and flexural characteristics of the nanocomposite compared to PPF alone [52]. Likewise, in a work carried out by Guo *et al*, a general and effective method for fabricating CNT–polymer fibers via the inclusion of monomers was achieved. This was followed by an *in situ* polymerization. The resulting nanocomposite demonstrated improved tensile strength compared pure CNT fibers and CNT–polymer fibers synthesized by the direct incorporation of polymers. Hence, this nanocomposite can be considered in the development of heart valves [53].

Zomer Volpato *et al* produced a MWCNT–polyamide 6 (PA6) nanocomposite and showed inclusion of up to 2 wt% CNTs in CNT–PA6 laminates; this enhanced the flexural stress of the laminates by up to 36%. The CNT was expected to form hydrogen bonds between the polymer and filler or form amide bonds among free amines on the polymer and the CNT’s carboxyl groups [54]. The physiochemical properties of PU have been improved by the inclusion of CNTs. For example, Amr *et al* showed that the Young’s modulus of CNT–polystyrene (PS) nanocomposites was improved by 22% [55]; Jung *et al* demonstrated that when the transparent PU film was added to functionalized MWCNTs, it displayed two- to ten-fold increases in tensile strength and Young’s modulus for MWCNT–PU composite film [56]. Tjing *et al* synthesized MWCNT–PU composites via electrospinning and solution casting techniques. The physical and thermal properties and mechanical performance of the nanofiber as well as the film composites were characterized and compared. The results showed that incorporation of MWCNTs improved the tensile strength and modulus of the composite nanofibers by 69% and 140%, respectively. On the other hand, for composite films the results were only 62% and 78% improvement in the tensile strength and modulus strength. The MWCNT–PU nanocomposite demonstrated an enhanced mechanical behavior with the addition of low MWCNT content. Hence, these studies demonstrate that the mechanical strength of the material improves tremendously with the inclusion of CNTs and this can be exploited for cardiovascular heart valve applications [57].

A few studies have shown that the mechanical characteristic of alumina can be reinforced by the addition of CNTs. As the mass fraction of CNTs was increased to 2.0 wt%, the tensile strength and Brinell hardness of the nanocomposite were improved and it attained a maximum of 245 MPa and 106.66 n mm^−2^, respectively. Ogihara *et al* developed a CNT–alumina nanocomposite by direct growth of CNTs on alumina via chemical vapor deposition and the nanocomposites were densified by spark plasma sintering. The mechanical strength was improved as follows: Young’s modulus, 383 GPa; Vickers hardness, 19.9 GPa; and bending strength, 578 MPa [58]. For zirconia–MWCNT composites, the inclusion of MWCNTs was expected to resist slow crack propagation and to improve the toughness of the ceramic material used for prostheses. The sample of zirconia–MWCNTs demonstrated a higher density, a smaller grain size, enhanced toughness and improved hardness, which shows the desirable characteristics of MWCNTs as bolstering agents for zirconia [59]. Hence all these results show that different matrices impregnated with CNTs improve the overall mechanical strength of the material, thereby warranting its potential for heart valve applications.

PVA hydrogels have been proposed as a viable biomaterial, yet these materials suffer from poor mechanical and water-retention properties. In work carried out by Zhang *et al*, the freeze–thaw method was utilized to add GO to PVA to yield GO–PVA nanocomposite hydrogels [60]. The mechanical properties of the GO–PVA hydrogels were significantly improved. In comparison to pure PVA hydrogels, a 132% improvement in tensile strength and a 36% enhancement of compressive strength were achieved with inclusion of 0.8 wt% of GO. This work demonstrates that excellent load transfer occurs between the GO and the PVA matrix, thereby suggesting that the same can be achieved in the development of heart valves with improved mechanical strength.

In work carried out by Jin *et al*, GO complexes with 2-(methacryloyloxy)ethyl phosphorylcholine (GO–MPC), as well as modified polyethylene GO–MPC (PE/GO–MPC) nanocomposites used for biomedical applications were produced and tested [61]. The results indicated that the tensile strength and elongation of the PE/GO–MPC nanocomposites were improved by 15.5% and 97.3%, respectively. Nanocomposite films of nanocrystalline cellulose acetate (NCCA) and GO were produced by Kabiri *et al* by combining NCCA and GO sheets in a well-controlled manner [62]. By the adjustment of GO content, various NCCA–GO nanocomposites with 0.3–1 wt% of GO were obtained. The preparation of nanocomposite films was performed using the solvent casting method. The characterization results of microscopic and XRD analysis showed that the GO nanosheets were uniformly dispersed in the NCCA matrix. The mechanical property of tensile strength of the resultant nanocomposites was studied. It was found that the best GO composition of the sample tested was 0.8 wt%, with a tensile strength of 157.49 MPa, where it demonstrated a 61.92% increase in tensile strength compared with plain NCCA. Thus, these studies validate the concept that these nanocomposites can be utilized to improve the mechanical strength of heart valves.

In work carried out by Zhang *et al*, an epoxy resin nanocomposite was added to 0.5, 1, 2 and 4 wt% pristine graphene to improve the mechanical properties of epoxy resin [63]. The modified GO nanoflakes were synthesized and utilized to create carbon fiber-reinforced and glass fiber-reinforced composite panels through a vacuum-assisted resin transfer molding process. The mechanical properties of the produced graphene composites were investigated as per the ASTM standards. It was found that the examined properties were enhanced consistently by an increase in the amount of nano inclusions. Specifically, in the case of 4 wt% GO in the resin, the tensile modulus, compressive strength and flexural modulus of the carbon fiber composites were enhanced from 15% to 21%, 34% to 84% and 40% to 68%, respectively. Similarly, the addition of 4 wt% pristine graphene in resin resulted in the improvement of the tensile modulus, compressive strength and flexural modulus of the carbon fiber composites from 11% to 7%, 30% to 77%, and 34% to 58%, respectively. Hence, the overall report suggests that the pristine graphene and modified GO nanoflake nanocomposite is a viable option to improve the mechanical strength which can be exploited for heart valves.

In a study carried out by Selvakumar *et al*, two types of 2D nHA rod (unmodified and polypropylene glycol (PPG)-wrapped) of different high aspect ratios were prepared using a modified co-precipitation method in the absence of any templates [64]. They were incorporated into a novel synthesized thermoplastic PU (TPU) matrix based on polycarbonate soft segments using *in situ* and *ex situ* techniques. High resolution transmission electron microscopy images of the prepared nHA and PPG–nHA crystals show that the mean particle length and width of the nanorods differs from one method to the other. The nanorods prepared using the PPG assisted method displayed a greater aspect ratio with tremendous uniformity in the length of individual rods. The average aspect ratio of the nanofiller was determined to be ∼4.8 for the nHA and ∼8.5 for the PPG-wrapped nHA. Moreover, it was found that the tensile strength, Young’s modulus and percentage of elongation of the nHA filled TPUs improved in comparison to pristine TPUs. The Young’s moduli of the nanocomposites were enhanced by 388%, 240%, 124% and 116% for *in situ* TPU–nHA, *in situ* TPU–PPG–nHa, *ex situ* TPU–PPG–nHA and *ex situ* TPU–nHA), respectively. In addition, the elongation at the break also improved for the nanocomposite systems. The prime reasons for the appreciable enhancement in properties are the high aspect ratio of nHA, the noncovalent surface modification of nHA, polymer–filler affinity, the surface roughness of the nHA rod and dispersion of the nanofiller. Hence, over all, higher aspect ratio (∼8.5) nHA filled TPU exhibits better properties. To conclude, the nanocomposites prepared using *in situ* techniques demonstrate better properties compared to the nanocomposites prepared using *ex situ* techniques. Hence, the novel TPU–PPG-wrapped HA nanocomposites can be used as promising biomaterials for heart valve development.

## 4. Nanocomposites for improving the anti-calcification potential of prosthetic heart valves

Calcification is the prime factor in the failure of a bioprosthesis such as a heart valve. It makes the heart valve vulnerable to structural failure and cusp tears. Bioprosthesis calcification is the process by which the mineral crystalline calcium phosphate and other calcium minerals deposit on the heart valve’s surface due to chemical reactions between aldehyde groups’ phospholipids and circulating calcium ions [65]. The general sites for calcific deposition are the commissural and basal areas of cusps where the mechanical stress exposure is highest in heart valves [65, 66]. In addition, several physiological parameters are also found to contribute to the calcification of heart valves [67]. Although many studies have been performed to develop a technique to minimize the calcification rate on heart valves, the issue has not yet been completely resolved [68–70]. Nanocomposites can be considered as a plausible candidate to solve the issue of calcification of heart valves.

**Figure 3.**
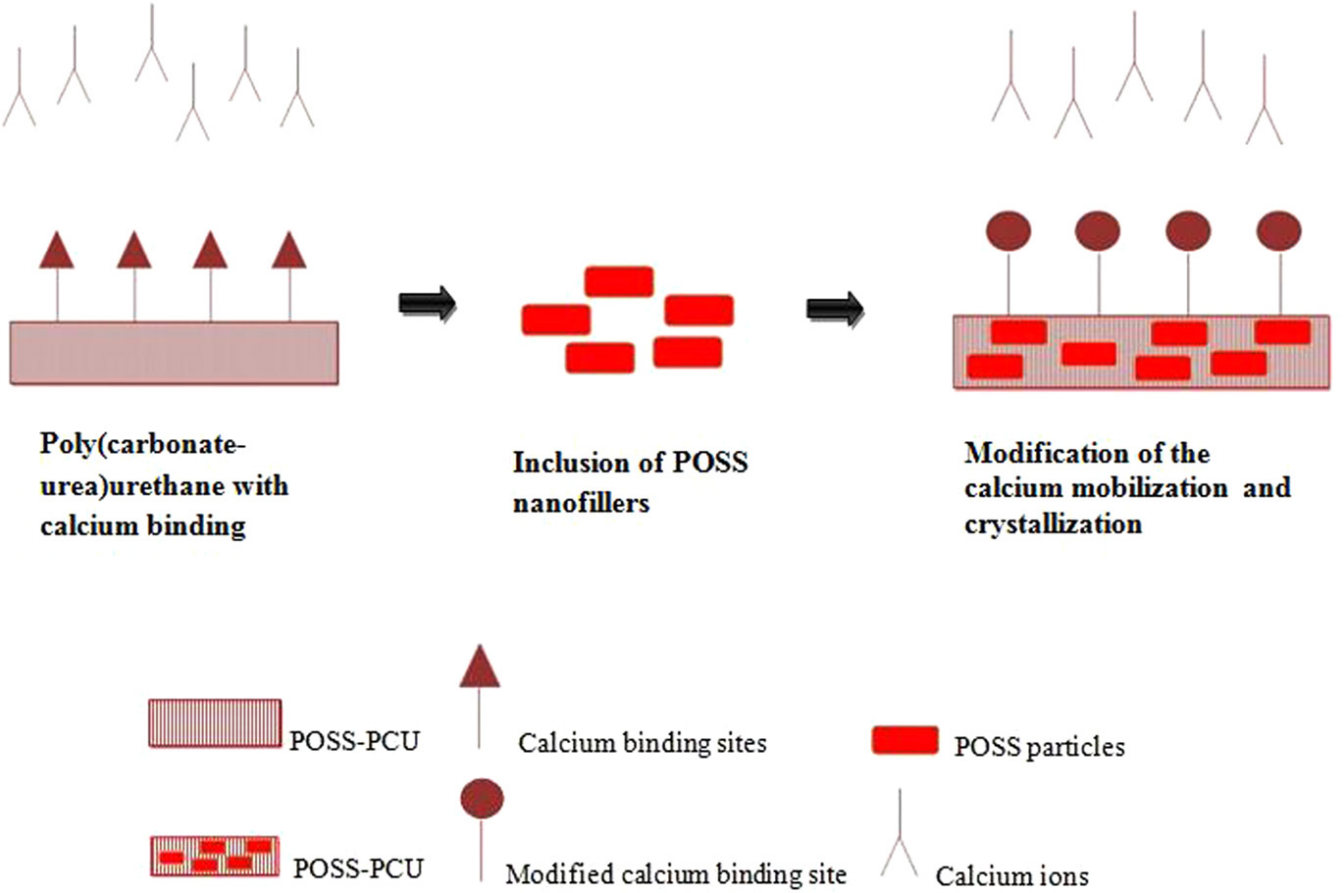
Anti-calcification properties enhanced by POSS inclusion in PCU.

In the work discussed previously [48], the anti-calcification potential of the silsesquioxane nanocomposite polymer was studied. In this work a novel nanocomposite polymeric material, POSS–PCU, was developed and investigated using an *in vitro* technique. Thin sheets of nanocomposite, glutaraldehyde-fixed bovine pericardium (BP) and PU were subjected to a calcium solution. The calcium solution was accelerated by an *in vitro* physiological pulsatile pressure system for a duration of 31 days for 4 × 10^7^ cycles. Then, the samples were tested for calcification using x-rays, microscopy techniques and chemical assessment. The mechanical and surface properties were determined using stress–strain behavior tests. The surface morphology and hydrophobicity were also studied. The results showed a difference in the calcification extent between the nanocomposite, glutaraldehyde-fixed BP and PU. It was found that glutaraldehyde-fixed BP and PU had appreciable calcium deposition but the calcium deposition on the nanocomposite was insignificant. In the calcified BP it was observed that the maximum calcification happened in regions of maximum mechanical stress in comparison to the peripheral regions near the clamping site. In the case of the POSS–PCU nanocomposite sample, it was noticed that there was no calcium deposition. However, quantitative analysis showed that there was only a meager amount of calcium present in the extracted solution. On the other hand, the PU samples had more calcium deposition, identified through x-ray and confocal microscopy studies. The quantitative analysis of the PU sample showed that the calcium present in the extracted solution was greater than that in the POSS–PCU sample (*p* < 0.05). This result may be interpreted as showing that the presence of POSS moieties in the PU made it more vigorous against calcification. Moreover, it is assumed that the POSS nanoparticles alter the surface calcium binding by modification of calcium mobilization and crystallization. This is illustrated in figure 3. The presence of POSS nanoparticles increases the hydrophobicity of the surface; thus it may be assumed that POSS has a repellent effect on mineral deposition. The result of this study was further supported by a study performed by Liao *et al*, where the mechanical stress exerted on the implanted heart valves played a vital role in onset and progression of calcification [71].

## 5. Nanocomposites for improving the hemocompatiblity of prosthetic heart valves

The hemocompatibility enhancement imparted by nanocomposites is highlighted in this section. A synthetic biomaterial experiences direct interaction with biological systems, specifically in an aqueous environment such as the cardiovascular system. It has an influence on peptide accumulation, mineral deposition and cell adhesion on the biomaterial’s surface. It was found that a hydrophobic material has better biocompatibility and lower cytotoxicity compared to a hydrophilic material which has an analogous formulation [72]. In addition, the hydrophobicity affects the protein adsorption and platelet activation of the biomaterial. For instance, the inclusion of hydrophobic phospholipid particles in the biomaterial’s surface has reduced propensity of biomaterials towards adsorption of bovine serum albumin and bovine plasma fibrinogen, and adhesion and aggregation of platelets [73].

Leitao *et al* performed a comprehensive blood compatibility investigation of the PVA–BC nanocomposite through the determination of whole blood clotting time, plasma recalcification, factor XII activation, platelet adhesion and hemolytic index [74]. The thrombogenicity and pro-coagulative activity of BC and PVA–BC were studied by determining the whole blood clotting time. ePTFE was used as a positive control and PS was utilized as a negative control. The BC and the nanocomposite failed to display significant differences, but they exhibited better values than the controls. Similarly, the plasma recalcification analysis also showed no improvement with the inclusion of PVA with BC; however, both samples showed improved properties compared with the controls. However, factor XII activation tests (intrinsic pathway) and complement system activation tests demonstrated improved activity of the PVA–BC nanocomposite compared with both pristine BC and the controls. Surprisingly, the extent of platelet adhesion was observed to be time dependent; initially, after 10 min ePTFE (a control) showed traces of platelet adhesion. BC and PVA–PC showed zero percentage adhesion in the beginning but after 50 min the same number of platelets were adhered as to ePTFE. This indicates that in both samples, platelets were activated only after 50 min. This process was meticulously analyzed by monitoring the signature of activated markers adhered to the platelets. Furthermore, the result of the decreased hemolytic index of the nanocomposite also suggests improved blood compatibility for the PVA–BC nanocomposite. Thus, the above results highlight the better rheological nature of the synthesized nanocomposite, which can be exploited for heart valve development.

The work undertaken by Kannan *et al* demonstrated the antithrombogenic nature of POSS–PCU, where protein and platelet adhesion were minimized [75]. It can be hypothesized that the surface constituent and morphology are the contributors to both the platelet and protein repellent activity. This was observed in POSS–PCU nanocomposites. These results are found to be in accordance with the findings of other researchers showing that POSS modified the surface of the PU layer by forming a silica layer on the surface of the PU which protects the POSS–PCU from further degradation [76]. Inclusion of POSS in PU improved the hydrophobicity of PU. It was found that the hydrophobicities of both PU and POSS–PCU were increased from the contact angle results, which increased due to exposure to the pulsatile flow system (*p* < 0.05). This may have been due to an interaction between the surface of the polymers and the electrolytes and ions present in the surrounding solution. Surface roughness has a significant effect on hydrophobicity. The Wenzel equation states that the contact angle on the surface is associated with the degree of ideal (true) contact and the roughness ratio (cos *θ*_W_ = *r* cos *θ*_i_). In this equation, *θ*_W_ is the Wenzel contact angle, where the apparent contact angle at the global energy minimum (GEM) *θ*_i_ is the ideal contact angle, and *r* is the roughness ratio which is defined as the ratio between the true and the apparent surface area of the solid. On a heterogeneous surface, the contact angle at the GEM is related to the ideal contact angle via the Cassie equation [77].

POSS–PCU displayed greater hydrophobicity than PU when it was exposed to a calcium solution. The effect of increased surface roughness and increased hydrophobicity in POSS–PCU compared to PU may have triggered the lower accumulation of calcium on the surface of the sample. Since the measured contact angle for POSS–PCU is greater than for PU, as per the Wenzel equation, the effect of changes in roughness can be greater for POSS–PCU than PU.

A MWCNT–PU composite was developed via controlled co-precipitation by Meng *et al* [78]. The surface chemical constitution of the treated CNTs was examined using x-ray photoelectron spectroscopy. Platelet adhesion and activation of the composite were determined using SEM and flow cytometric analysis, respectively, and the problems associated with red blood cells were evaluated through the measurement of the absorbance of free hemoglobin. The results of the study show that MWCNTs with oxygen-containing functional groups were evenly dispersed in the PU matrix via controlled co-precipitation and the resultant nanocomposite surface displayed an appreciably enhanced anticoagulant function, which validates its viability in serving as a putative material in the development of cardiovascular heart valves.

For heart valve applications, the design of a hemocompatible surface is required to reduce platelet surface interactions and to improve the thromboresistance of the surface. In a study by Dhandayuthapani *et al*, SWCNTs and zein fibrous nanocomposite scaffolds were combined by electrospinning and the antithrombogenicity and hydrophilicity were analyzed [79]. Uniform and highly smooth nanofibers of zein nanocomposite with different SWCNT contents (ranging from 0.2 wt% to 1 wt%) were prepared using an electrospinning technique without the occurrence of bead defects. The resultant fiber diameters were 100–300 nm without any beads. The characterization of the composite nanofibers with and without SWCNTs was performed using SEM, transmission electron microscopy, thermogravimetric analysis and tensile mechanical testing. The hemolytic and platelet adhesion characteristics of the nanocomposite zein–SWCNTs were determined. The results showed that zein–SWCNT composite scaffolds can be considered as a putative antithrombotic material and a promising biomaterial for heart valve applications.

In a study undertaken by Jin *et al*, a range of functional GOs based on the biomimetic monomer 2-(methacryloyloxy) ethyl phosphorylcholine (GO–g-pMPC) were synthesized by reverse atom transfer radical polymerization in an alcoholic medium using peroxide groups as the initiator [80]. This was then filled into the PU matrix to obtain PU/functional GO nanocomposite film (PU/GO–g-pMPC). The results showed that the inclusion of a very small amount of GO can improve the hemocompatible properties of PU. The hemocompatiblity of the PU substrates was examined by protein adsorption tests and platelet adhesion tests. The result showed that all the PU/GO–g-pMPCs displayed enhanced resistance to nonspecific protein adsorption and platelet adhesion.

Carboxylated GO (GO–COOH) with glutamic acid (Glu) was prepared at different pH values by Zhou *et al* [81]. GO–COOH/Glu was characterized by Fourier transform infrared spectroscopy, XRD, thermogravimetry and zeta potential measurements followed by blood compatibility analysis. The results indicate that the complexes have a close relationship with pH value owing to the presence of acid-responsive GO–COOH/Glu complexes with Glu through amidation reactions in the basic domain. The hemocompatiblity of GO–COOH/Glu complexes was studied via hemolysis and recalcification tests. The results showed that the plasma recalcification time was delayed greatly in whole blood, and the hemolysis rates were lower than 5%. Thus, GO–COOH/Glu complexes are hemocompatible and this nanocomposite can be used as a cardiovascular heart valve material.

In a study performed by Lee *et al*, the hemocompatibility of a graphene–heparin conjugate was determined via noncovalent interaction between chemically reduced graphene and heparin [82]. The negative charge of heparin on graphene plates enables the hydrophobic graphene to be solubilized in aqueous media with the absence of precipitation or aggregation, even after six months. Unfractioned heparin (UFH) with a high molecular weight was efficient for graphene solubilization whilst low molecular weight heparin was poor for graphene solubilization. Noncovalent interaction of heparin chains on graphene plates conserves their anticoagulant activity even after conjugation with graphene. Graphene–UFH conjugate displays an improved anti-factor Xa (FXa) activity of 29.6 IU mL^−1^ compared with pristine GO (1.03 IU mL^−1^) which can be exploited for cardiovascular heart valve applications.

Carbon nanofibers (CNFs) embedded in poly(lactic-co-glycolic-acid) (PLGA) were found to promote cardiomyocyte growth compared to the conventional polymer substrate and the mechanism involved was also studied [83]. In this work, CNFs were added to biodegradable PLGA (50:50 PGA:PLA weight ratio) to improve the conductivity, and mechanical and cytocompatibility properties of pure PLGA. Different PLGA to CNF ratios (100:0, 75:25, 50:50, 25:75 and 0:100 wt%) with varying PLGA densities (0.1, 0.05, 0.025 and 0.0125 g mL^−1^) were utilized, and their compatibility with cardiomyocytes was studied. Among different cytocompatibility tests, it was found that cardiomyocytes were viable and expressed vital biomarkers, such as cardiac troponin T, connexin-43 and alpha-sarcomeric actin. Adhesion and proliferation tests displayed a PLGA density of 0.025 g mL^−1^ with PLGA to CNF ratios of 75:25 and 50:50 (wt%) promoting the best overall cell growth, which is a 55% improvement in cardiomyocyte density after 120 h in comparison to pure PLGA and a 75% improvement in cardiomyocyte density compared to the control at the same time point for 50:50 (wt%). AFM revealed that the addition of CNFs to PLGA increased the material surface area from 10% (100:0; PLGA to carbon nanofiber (wt%:wt%)) to more than 60% (50:50; PLGA to carbon nanofibers (wt%:wt%)). Furthermore, the adsorption of specific proteins such as fibronectin and vitronectin demonstrated more adsorption for the 50:50 PLGA to CNF (wt%:wt%) ratio at 0.025 g mL^−1^ PLGA in comparison to pure PLGA, and shows that the cardiomyocyte function increased on CNF-enriched composites. Thus, this work indicates that cardiomyocyte function was improved with 50:50 PLGA to CNF (wt%:wt%) composite ratios at 0.025 g mL^−1^ PLGA densities since they mimicked native heart valves and improved the adsorption of proteins known to promote cardiomyocyte function.

As a continuation to the previously discussed study by Selvakumar *et al*, the optical densities of MG63 cell proliferation on the surfaces of pristine TPU and nanocomposites were studied after 1, 5 and 7 days [64]. Cell proliferation was noticeable over time. This suggests that more favorable cell growth was observed on the surface of *in situ* TPU–PPG–nHA nanocomposites compared to the nanocomposites prepared via an *ex situ* technique such as TPU–nHA. This is mainly due to the interfacial adhesion between the nHA and TPU matrix. The field emission SEM images of MG63 cells cultured and fixed after 7 days show that cell spreading and cellular processes are improved on the nHA filled TPU nanocomposite surface. Either the prothrombin time (PT) or activated partial thromboplastin time (APTT) of the nanocomposites samples were increased in comparison to the pristine TPU. Statistical analysis of the pristine TPU performed using one-way ANOVA indicates appreciable differences (*p* < 0.05) between TPU and the nanocomposites for both PT and APTT times. The hemolysis assay showed that pristine TPU triggered 12% hemolysis; however the nanocomposite samples demonstrated less than 1% hemolysis. Specifically, *in situ* prepared samples showed only 0.3% hemolysis, and this shows that antithrombotic activity is exceptionally good for such nanocomposites. Thus, they can also be described as nonhemolytic materials. To conclude, nHA filled TPU composites increase the time taken for blood coagulation and also minimizes the hemolysis ratio greatly, specifically for *in situ* prepared nanocomposites of both fillers (nHA and polymer wrapped nHA). Different factors determining the blood compatibility are the degree of hydrophilicity, the surface roughness of filler and of course the biocompatibility of nHA, etc. Hence, these novel nanocomposites can be utilized for blood interacting applications because of their favorable hemocompatibility as well as their excellent antithrombotic properties. Thus, we envisage that the novel TPU–PPG-wrapped HA nanocomposites hold great potential for biomedical applications, especially for cardiovascular heart valves.

## 6. Discussion

**Figure 4.**
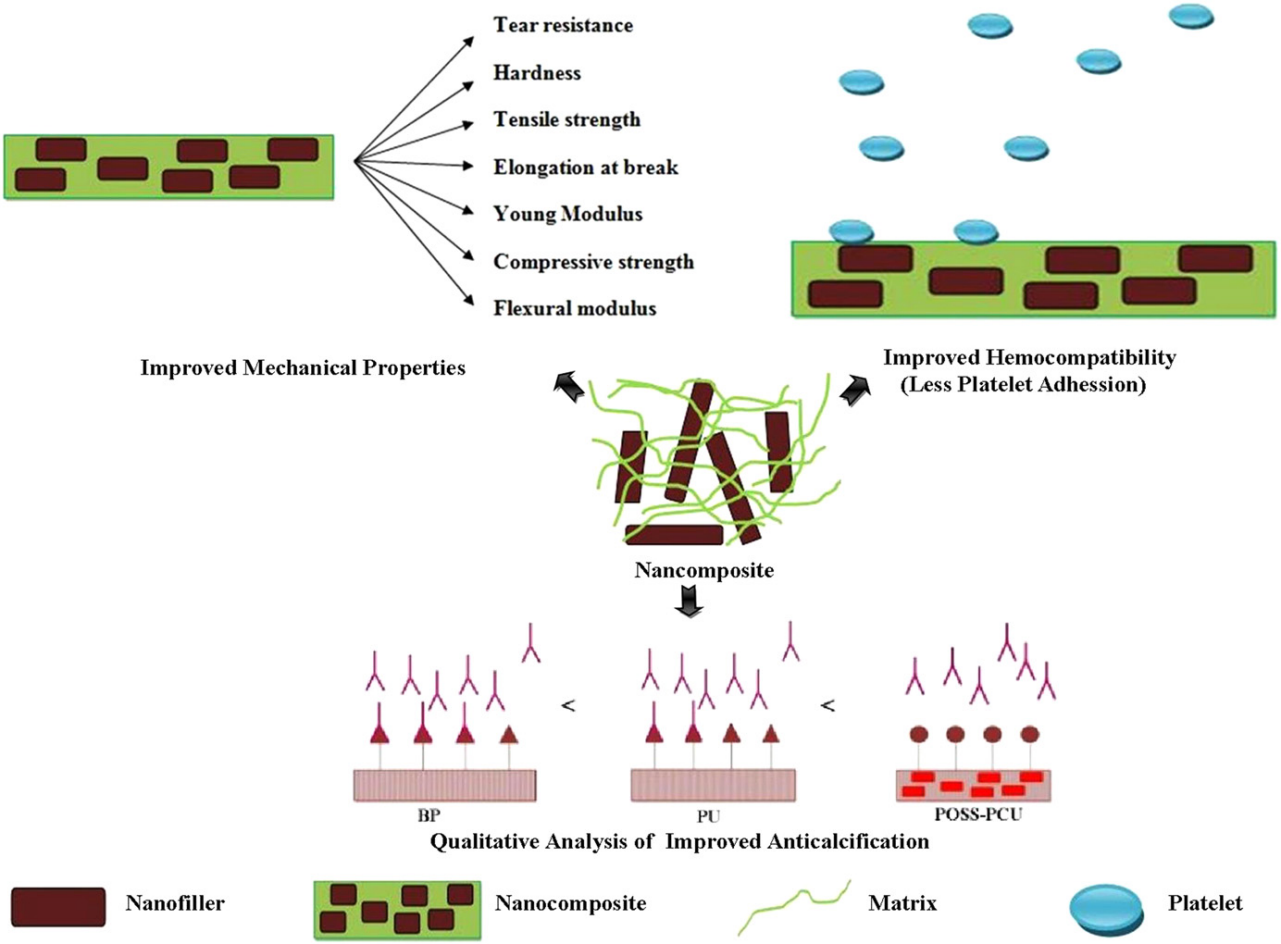
Properties enhanced by nanocomposites for heart valve applications.

Nanocomposites with excellent properties have been introduced with the latest advancements in nanotechnology. These nanocomposites can be considered for various biomedical applications, particularly cardiovascular applications, which are currently in high demand. The results of each study performed on different nanocomposites along with their key properties are collected in table 1. Figure 4 illustrates the properties enhanced by the nanocomposites for heart valve applications. From the thorough analysis of these different studies, it can be concluded that the MWCNT–PU, GO–PVA and POSS–PCU nanocomposites are the most promising for bolstering the mechanical strength of prosthetic heart valves. This is because the inclusion of MWCNTs was shown to improve the tensile strength and modulus of the composite nanofibers by 69% and 140%, and produce a ten-fold increase in Young’s modulus. GO incorporation with PVA also resulted in a 132% improvement in tensile strength and a 36% enhancement of compressive strength with the inclusion of 0.8 wt% of GO in PVA. Despite possessing good mechanical properties, nanocomposites are the focus of debate regarding their potential toxicity and few reviews have been published on this topic [84–89]. For instance, in a recent work, CNTs were shown in some cases to induce genotoxic effects [90]. However, another *in vivo* study of the biological response to a CNT network was performed in the zebrafish model [91]. It was found that while pristine CNTs had been previously found to exert genotoxic effects *in vitro*, the CNT network was not genotoxic *in vivo*. Thus, a consensus has yet to be achieved because conflicting results have been obtained in toxicology studies [92, 93] The lack of standardized protocols as well as the variability of nanocomposites used in different studies is the main reason for this discrepancy [92–98]. In fact, there is also evidence that shows that the responses of cells to nanofillers such as CNTs are modulated by their physico-chemical properties and functionalization [99, 100]. Hence, looking at this evidence from a different viewpoint, the possibility of reducing the toxicity of some nanofillers such as CNTs and GO by modifying their physico-chemical properties circumvents the problems of designing safe CNTs and GO, opening up their prospects for secure and improved nanocomposite heart valves.

**Table 1.** Nanocomposites used for cardiovascular heart valve applications.

Study No.	Matrix material	Nanofiller	Key properties	Reference
1	Bacteria culture media	Hydroxyapatite (HA) powder, carboxymethylcellulose (CMC) and nano-hydroxyapatite (nHA) nanoparticles	An increase in the mechanical strength of the resultant nanocomposite.	[44]
2	Polyvinyl alcohol (PVA)	Bacterial cellulose (BC)	Improves the mechanical strength of the biocompatible material.	[45]
			A broader range of mechanical properties and higher strength.	[65]
			The blood compatibility of the PVA–BC nanocomposite was improved.	[74]
3	Poly(carbonate-urea)urethane (PCU)	Polyhedral oligomeric silsesquioxane (POSS)	Improved tensile strength, tear resistance, hardness, elasticity properties, tear resistance and hardness.	[46]
			The strength and hardness of the PCU increased due to the inclusion of POSS particles.	[47]
			A strong framework with shorter bond lengths exists between nanocomposites providing them with additional resistance to degradation.	[47]
			Enhancement of creep and compression set properties.	[15]
			The maximum tensile strength and elongation at the break were found to be smaller in the PU than the POSS–PCU.	[48, 49]
			Calcification occurred in regions of maximum mechanical stress in comparison to the peripheral regions near the clamping.	[71]
			POSS nanoparticles increase the hydrophobicity of the surface, thus it may be assumed that POSS has a repellent effect on mineral deposition.	[58]
			Protein and platelet adhesion was minimized.	[75]
			A silica layer on the surface of PU protects the POSS–PCU from further degradation.	[76]
			Inclusion of POSS in PU improved the hydrophobicity of PU.	[76]
			The roughness of the surface increases the contact angle.	[77]
4	Poly(lactic-co-glycolic-acid) (PLGA)	Carbon nanofibers (CNFs)	Cardiomyocyte function increased on CNF-enriched composites.	[83]
5	Propylene fumarate (PPF)	Single-walled carbon nanotubes (SWCNTs)	The inclusion of SWCNTs in the PPF polymer improves the mechanical properties of the PPF.	[51]
			Very low concentrations of SWCNTs in the PPF polymer matrix improve the compressive and flexural characteristics of the nanocomposite up to two- to threefold compared to PPF alone.	[52]
6	Polymer fibers	Carbon nanotubes (CNTs)	Improved tensile strength compared to pure CNT fibers or the CNT–polymer fibers synthesized.	[53]
7	Polyamide 6 (PA6)	Multi-walled carbon nanotubes (MWCNTs)	Enhanced storage modulus and loss modulus of the CNT–PA6 nanocomposite.	[54]
			Inclusion of up to 2 wt% CNTs in CNT–PA6 laminates enhanced the flexural stress of the laminates up to 36%.	[54]
8	Polystyrene (PS)	CNTs	Young’s modulus of CNT–PS nanocomposites was improved by 22%.	[55]
9	Polyurethane (PU)	MWCNTs	Two-fold and ten-fold increases in tensile strength and Young’s modulus.	[56]
			The inclusion of MWCNTs improved the tensile strength and modulus of the composite nanofibers by 69% and 140%.	[57]
10	Alumina	CNTs	The resultant nanocomposite surface displayed appreciably enhanced anticoagulant function.	[78]
			The mechanical strength was improved as follows: Young’s modulus, 383 GPa; Vickers hardness, 19.9 GPa; and bending strength, 578 MPa.	[58]
11	Zirconia	MWCNTs	Higher density, smaller grain size, enhanced toughness and improved hardness.	[59]
12	Zein fibers	SWCNTs	Improved hemolytic and platelet adhesion characteristics.	[79]
13	Polycarbonate based PU	nHA	Nanorods prepared using a PPG assisted method displayed a greater aspect ratio and tremendous uniformity.	[64]
			The tensile strength, Young’s modulus and percentage of elongation of the nHA filled TPUs improved.	[64]
			Cell spreading and cellular processes are improved for nHA filled TPU.	[64]
			Prothrombin time (PT) and activated partial thromboplastin time (APTT) are increased.	[64]
			Hemolysis of nanocomposite samples demonstrated less than 1% hemolysis.	[64]
14	PVA	Graphene oxide (GO)	A 132% improvement in tensile strength and a 36% enhancement of compressive strength were achieved with inclusion of 0.8 wt% of GO.	[60]
15	2(methacryloyloxy)ethyl phosphorylcholine (GO–MPC)	GO complexes	The tensile strength and elongation of PE/GO–MPC nanocomposites were improved by 15.5 and 97.3%.	[61]
16	Nanocrystalline cellulose acetate (NCCA)	GO	A 61.92% increase in tensile strength compared with plain NCCA.	[62]
17	Epoxy resin	0.5, 1, 2 and 4 wt % pristine graphene	In the case of 4 wt % GO in the resin, the tensile modulus, compressive strength and flexural modulus of carbon fiber composites were enhanced by 15% (21%), 34% (84%) and 40% (68%), respectively.	[63]
18	2-(methacryloyloxy) ethyl phosphorylcholine and PU(PU/GO–g-pMPC)	GO	Inclusion of a very small amount of GO can improve the mechanical properties of PU.	[80]
			Protein adsorption test and platelet adhesion test results show improved hemocompatibility.	[80]
19	Glutamic acid (Glu)	Carboxylated GO (GO–COOH)	Recalcification time was delayed greatly in the whole blood and the hemolysis rates were lower than 5%.	[81]
20	Unfractioned heparin (UFH)	Graphene	Graphene–UFH conjugate displays an improved FXa activity of 29.6 IU mL^−1^.	[82]

The anti-calcification effect was observed most in the case of POSS–PCU compared to other available nanocomposites because the POSS nanoparticles increase the hydrophobicity of the surface; thus it may be assumed that POSS may have a repellent effect on mineral deposition. However, the role of other nanocomposites such as CNTs, GO and BC should not be ignored as the studies related to the anti-calcification potential of these composites have not been analyzed exhaustively. Hence, it is recommended that some comparative trials of the various nanocomposites are initiated in order to obtain a better understanding of anti-calcification potential. In the case of nHA, there is a general perception that it may exhibit calcification. However, the works utilizing nHA as a filler discussed in this review showed that the inclusion of only 1 wt% in the matrix polymer was found to have promising results in improving the mechanical as well blood compatibility properties of the matrix material [64]. In fact, in a recent work performed by Chen *et al*, poly(*∊*-caprolactone) nanofibers with similar diameters (340 ± 30 nm) but different nHA concentrations (0–50%) were fabricated and the effect of the nHA concentration on mineralization was investigated. The mineralization extent was found to be highest in nanofibers with 50% nHA [101]. Another *in vivo* work shows that the apatite mineral crystals are deposited most on the polymer–HA nanocomposites with higher nHA nanofiller content [102]. Hence, when the inclusion of nHA is at a very low quantity such as 1 wt%, as discussed, this may not pose a significant threat of calcification.

From the blood compatibility enhancement studies, it was found that CNFs embedded in PLGA can be a plausible candidate since cardiomyocyte function increases on CNF-enriched composites. Likewise, the exhaustive analysis of works performed using POSS–PCU shows that this nanocomposite may have good blood compatibility. This is because protein and platelet adhesion was minimized in POSS–PCU, which also had an increased surface roughness. Moreover, CNTs or CNFs embedded in a biodegradable polymer exhibit biocompatibility even after release into blood vessels. The work carried out by Sitharaman *et al* showed that the *in vivo* biocompatibility of ultra-short SWCNT/PPF nanocomposites is similar to that of PPF alone, and the tissue response they elicit is similar to that of other polymers and nanocomposites used for tissue engineering [52, 103–106]. Hence, it is anticipated that CNTs or CNFs embedded in biodegradable polymers do not pose a threat to biocompatibility. Similarly, in work carried out by Edwards *et al* MWCNT yarn and a composite scaffold of MWCNT/PLGA, formed by electrospinning PLGA nanofibers, were investigated for *in vitro* biocompatibility with NR6 mouse fibroblast cells for up to 22 days. The results indicated that even the MWCNT yarn supported cell growth throughout the culture period, with fibroblasts attaching to and proliferating on the yarn surface, indicating its biocompatibility [107].

In addition to this, the problem of biodegradability as well as biocompatibility can be addressed by functionalization of CNTs. Different approaches have been developed to make CNTs biocompatible and to modulate any ensuing toxic effects. Although chemically functionalized CNTs display reduced toxicity, they are still considered with skepticism owing to their perceived non-biodegradability. In a recent work, it was demonstrated that functionalized CNTs can be degraded by oxidative enzymes [99]. In addition, the biocompatibility and low cytotoxicity of CNTs are attributable to size, dose, duration, testing systems and surface functionalization. The functionalization of CNTs enhances their solubility and biocompatibility and modifies their cellular interaction pathways, resulting in much-reduced cytotoxic effects and improved biocompatibility [100]. Thus, when functionalized CNTs are used for heart valves, they will exhibit biocompatibility even after release into blood vessels due to the degradation of polymers.

## 7. Conclusions

**Figure 5.**
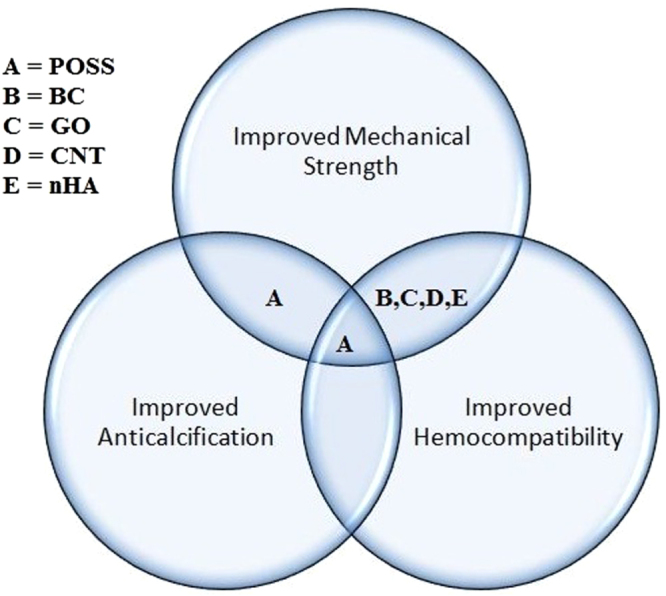
Properties enhanced by inclusion of nanofillers in the resultant nanocomposites.

Recently, nanocomposites utilizing POSS–PCU, BC, CNTs, GO and nHA have been explored for heart valve applications. Desirable properties are enhanced by the inclusion of nanofillers in the resultant nanocomposites, shown in figure 5. It was found that these nanocomposites can play a key role in improving heart valve mechanical strength, anti-calcification potential and hemocompatibility. However, which nanocomposite offers the best potential for heart valves is still largely a matter of conjecture. This is due to the lack of studies of different nanocomposites with identical experimental and clinical settings. There are still vital challenges such as material costs, design and processing, longevity, and health and safety approvals to tackle, in order to establish the functionality and efficiency of these novel nanocomposites as heart valve materials. We anticipate that synergetic efforts of engineers, biologists and surgeons will be required to design clinically available nanocomposite heart valves.
